# Characteristics and Associated Risk Factors of Neonatal Sepsis: A Retrospective Study From Saudi Arabia

**DOI:** 10.7759/cureus.76517

**Published:** 2024-12-28

**Authors:** Hussain A Al Ghadeer, Rahmah H Alabdallah, Ghufran I AlKhalaf, Faisal K Aldandan, Hassan A Almohammed, Murtadha M Al Busaeed, Fatimah m Alkhawajah, Khawla A Al Hassan, Fatimah A Alghadeer, Haidar H Alreqa, Rafyel S Al Muaiweed, Afnan S Al Bohassan, Abdullatif A AlMuhaish, Ahmed J Alhabeeb, Anas k Alsaif

**Affiliations:** 1 Pediatrics, Maternity and Children Hospital, AlAhsa, SAU; 2 Neonatology, Maternity and Children Hospital, AlAhsa, SAU; 3 Infectious Disease, Maternity and Children Hospital, AlAhsa, SAU; 4 Pediatrics, King Faisal University, AlAhsa, SAU; 5 Pharmacology, King Faisal University, AlAhsa, SAU; 6 Pediatrics, Almaarefa University, Riyadh, SAU; 7 Pharmacology, Ministry of National Guard, AlAhsa, SAU

**Keywords:** alahsa, antibiotic resistance, antibiotic sensitivity, neonatal intensive care unit, neonatal sepsis, prevalence, risk factors, saudi arabia, septicemia

## Abstract

Introduction

Neonatal sepsis is defined as a systemic illness caused by bacteria, viruses, or fungi, characterized by hemodynamic abnormalities and clinical findings that result in morbidity and mortality. Neonatal morbidity and mortality are significantly influenced by neonatal sepsis. Causative pathogens and antimicrobial sensitivity profiles have changed over time, with significant geographic variation.

Aim

To determine the characteristics and associated risk factors of sepsis among neonates admitted into neonatal intensive care units (NICU) in Maternity and Children Hospital, AlAhsa, Saudi Arabia.

Methodology

An institution-based retrospective cross-sectional study was conducted among neonates who were admitted to the neonatal intensive care unit from January 2022 to December 2023 at the Maternity and Children Hospital, AlAhsa, Saudi Arabia. All neonates born with clinically diagnosed sepsis and positive culture were included in this study.

Results

This study included 134 neonates with a culture-proven diagnosis of neonatal sepsis during the study period. There were 23 (17.2%) cases of early-onset sepsis (EOS) and 111 (82.8%) cases of late-onset sepsis (LOS). Compared to late-onset sepsis (18.8%), *Pseudomonas aeruginosa* is more common in early-onset sepsis (33.3%). In cases of early-onset sepsis, *Escherichia coli* is isolated more often (33.3%) than in cases of late-onset (9.4%). Neonatal sepsis mortality is higher in LOS (25 [22.5%]) than in EOS (3 [13%]). Neonates with extremely preterm birth weight, gram-negative sepsis, and thrombocytopenia have a significantly higher mortality rate (p=<0.05).

Conclusion

In order to lower the risk among newborns, policymakers and/or managers will benefit from the information provided by the assessment of the prevalence, clinical outcomes, and risk factors for neonatal sepsis. Furthermore, developing hospital-based care strategies requires an understanding of the microorganisms that cause infections among neonates.

## Introduction

Neonatal sepsis is a syndrome characterized by the clinical presentation of inflammatory responses resulting from a variety of systemic infections, including meningitis, septicemia, pneumonia, and infections related to the bones. Sepsis's non-specific clinical signs and symptoms mimic a variety of illnesses unrelated to infections. Nonetheless, in spite of this predicament, there is still a lack of global guidelines and consensus definitions because of variations in epidemiologic patterns and clinical practices. While blood culture is widely regarded as the gold standard for diagnosing neonatal sepsis, low positivity rates represent a significant management challenge [1-3].

Early-onset sepsis (EOS) occurs at less than 72 hours of age, while late-onset sepsis (LOS) occurs at more than 72 hours of age. These classifications are based on the presentation of the disease and have implications for potential risk factors, likely organisms, and suggested empirical treatments [4]. Together, group B *Streptococcus* and *E. Coli* are responsible for about 70% of cases of early-onset neonatal sepsis [5]. On the other hand, Gram-positive organisms were responsible for 70% of late-onset infections, with coagulase-negative *Staphylococci*, *Salmonella*, and *Listeria monocytogenes* accounting for about 48% of the infections [6].

Neonatal sepsis was predisposed to by a number of factors, including low APGAR score at minute five, residence of delivery, premature rupture of membranes, intrapartum fever, and delayed crying at birth. While prematurity, low birth weight, residence, parity, antenatal care services, mode of delivery, and alcohol with offensive odors are acknowledged as potential independent risk factors of neonatal sepsis [7, 8]. Once sepsis is suspected, empirical antibiotics are typically started. However, the growing number of multidrug-resistant organisms limits the available antibiotics and delays the implementation of appropriate treatments [8]. For this reason, institutional guidelines based on the prevalence of microorganisms in a given area and their patterns of antibiotic susceptibility are required [9]. When sepsis or septic shock is diagnosed, empirical intravenous antibiotics should be started within the first hour, per the guidelines of the Surviving Sepsis Campaign (SSC) [10-12].

Sepsis remains one of the leading causes of morbidity and mortality among newborns worldwide, even with advancements in the healthcare system. In 2018, 2.5 million neonates died. The levels and trends in child mortality report (2019) state that neonatal mortality rates vary greatly amongst global regions. According to a systematic review evaluating the prevalence of sepsis in newborns and children worldwide, 20% of affected cases may die from severe sepsis [13]. Sub-Saharan Africa and South Asian countries have been found to have the highest rates of neonatal mortality [14]. Neonatal mortality rates are estimated to be 20 per 1000 live births in low- and middle-income countries (LMICS) and three per 1000 in high-income countries [15]. Compared to 6% in developed countries, neonatal sepsis accounts for 17% of deaths in Sub-Saharan Africa [16].

A number of studies were carried out to determine the epidemiology of neonatal sepsis in the various regions of the Kingdom of Saudi Arabia. Prior to the year 2000, *Staphylococcus epidermidis* was the most frequently isolated pathogen from neonates admitted to tertiary care hospitals in Riyadh [17] and Khobar [18] after they developed sepsis. Similar results were found in newborns admitted to King Khalid University Hospital in Riyadh between 1999 and 2007 who had low birth weights (between 500 and 1500 grams) [19]. A recent study in Riyadh [20], Jeddah [21], and *Almadenia* [22] showed that the most reported organism is group B *Streptococcus*, followed by *Escherichia coli*.

The type of microorganism, the onset of sepsis, the site of infection, and other risk factors for sepsis all influence the prognosis significantly. Accurate monitoring and evaluation of these parameters are crucial for improving prognosis and lowering the mortality rate in neonatal sepsis. Thus, the purpose of this study is to investigate the prevalence and risk factors of neonatal sepsis, as well as the causal bacteria and their antibiotic susceptibility profiles.

## Materials and methods

Aim of the study

The aim of this study is to determine the prevalence and associated risk factors of neonatal sepsis as well as the causative microorganisms and their antimicrobial susceptibility profile.

Rationale and importance

The effective implementation of appropriate antimicrobial therapy guidelines is crucial for controlling antimicrobial resistance. Continuous surveillance of the microbiological etiologies of neonatal sepsis and their antimicrobial sensitivity patterns is necessary for every hospital. This could lead to both a decrease in mortality from sepsis and an increase in the use of affordable treatments.

Study design and population

The maternity and children's hospital in AlAhsa, Saudi Arabia, is the site of this descriptive retrospective study from January 2022 to December 2023, which reviewed the medical records of mothers and their newborns who were diagnosed with sepsis. A single pathogenic organism growing in blood, cerebrospinal fluid, or urine combined with laboratory and clinical evidence of infection is considered sepsis. Any proven positive cultures (blood, urine, CSF) among neonates (age ≤28 days old) were included in the study. Negative cultures and neonates >28 days old were excluded from the study. 

Data collection

The demographics of the mothers and their newborns, as well as perinatal clinical factors like gestational age, delivery method, pregnancy-related complications, complete blood count, isolated organisms, antibiotic use, and length of hospital stay, are all recorded on a data extraction sheet. Blood cultures were taken from every patient upon admission before starting antimicrobial therapy. A total of 2 milliliters of blood was extracted via a unilateral venipuncture in an aseptic technique. After five days of incubation, blood cultures that showed no signs of microbial growth were considered negative. Positive samples were grown on chocolate agar, MacConkey, and blood cultures. The plates were incubated at 37°C for 24 to 48 hours before being checked for growth. The clinical microbiology procedures handbook [23] states that the morphology of the colony, Gram staining, and analysis of biochemical properties are used to identify organisms.

Data analysis

The data were collected, reviewed, and then inserted into IBM Corp. Released 2019. IBM SPSS Statistics for Windows, Version 26.0. Armonk, NY: IBM Corp. All statistical methods used were two-tailed with an alpha level of 0.05, considering significance if the p-value was less than or equal to 0.05. Descriptive analysis for categorical data was done using frequencies and percentages, whereas numerical data were presented as means with standard deviations. Also, neonates' bio-demographic data, sepsis data, isolated organisms, laboratory findings, and clinical outcomes were tabulated and graphed. Cross tabulation for showing factors associated with the neonates' clinical outcome using the Pearson chi-square test and exact probability test for small frequency distributions.

## Results

Table 1 shows a total of 134 eligible NICU-admitted neonates with neonatal sepsis were included. Seventy-seven (57.5%) neonates were males, with single pregnancies for 123 (91.8%) while 11 (8.2%) came with multiple pregnancies. A total of 41 (30.65%) neonates were extremely preterm, 28 (20.95%) were very preterm, and 33 (24.7%) were moderate to late preterm. As for birth weight, 38 (28.4%) had extremely low birth weight, 31 (23.1%) had very low birth weight, and 32 (23.9%) had low birth weight. A total of 73 (54.5%) neonates were born by normal vaginal delivery.

**Table 1:** Bio-demographic characteristics of the study NICU-admitted neonates with neonatal sepsis, Al-Ahsa, Saudi Arabia (n=134)

Bio-demographics	No	%
Gender		
Male	77	57.5%
Female	57	42.5%
Gestation		
Single	123	91.8%
Twin	8	6.0%
Triple	3	2.2%
Gestational age (By weeks)		
Extreme preterm (<28 W)	41	30.6%
Very preterm (28 to <32 W)	28	20.9%
Moderate preterm (32 to <34 W)	12	9.0%
Late preterm (34 to <37 W)	21	15.7%
Full term (37 to 42 W)	32	23.9%
Birth weight (By grams)		
ELBW (<1000 g)	38	28.4%
VLBW (1000 to 1499 g)	31	23.1%
LBW (1500 to 2499 g)	32	23.9%
NBW (2500 to 3999 g)	33	24.6%
Mode of delivery		
Cesarean section	61	45.5%
Vaginal delivery	73	54.5%

Table 2 shows the neonatal sepsis clinical data and associated organism isolation among NICU-admitted neonates with neonatal sepsis. About the onset of sepsis, it started early (≤ 72 hours) among 23 (17.2%) neonates, but among most of them (82.8%), sepsis started late after 72 hours. A total of 63 (47%) neonates had critical conditions. As for infection type, it was bacterial among the vast majority of the neonates (85.1%; 114), mainly gram-negative (40.2%) and gram-positive (40.2%), fungal among 12 (9%), and 8 (6%) had both types. A total of 99 (73.9%) neonates had single organisms isolated, and 35 (26.1%) had multiple organisms isolated. The isolated peripheral blood organisms were mainly of a single type (87.5%), versus all urine and CSF samples. At the trachea, one case had 3-5 organisms isolated, and in a central blood sample, one case had three isolated organisms. A total of 54 (40.3%) cases had thrombocytopenia (<150), and 41 (30.6%) had leukopenia (less than 5000).

**Table 2:** Neonatal sepsis clinical data and associated organism isolation among NICU-admitted neonates with neonatal sepsis, Al-Ahsa, Saudi Arabia (n=134)

Sepsis	No	%
Onset of sepsis		
Early (≤ 72 hours)	23	17.2%
Late (>72 hours)	111	82.8%
The clinical condition of the baby		
Critical (Mechanical ventilation, inotropic support...etc.)	63	47.0%
Stable (Room air, non-invasive ventilation, feeder and grower...etc.)	71	53.0%
Type of infection		
Bacterial	114	85.1%
Gram-negative	57	46.7%
Gram-positive	49	40.2%
Both types	16	13.1%
Fungal	12	9.0%
Both (Bacterial and Fungal)	8	6.0%
Isolated organism number		
Single	99	73.9%
Multiple	35	26.1%
2-3	30	22.4%
4-5	4	3.0%
6+	1	.7%
Blood Peripheral line		
1 organism	88	81.5%
2 organisms	13	12.0%
3-5 organisms	7	6.4%
Blood central line		
1 organism	7	87.5%
3 organisms	1	12.5%
Urine		
1 organism	4	100.0%
CSF		
1 organism	4	100.0%
Eye		
1 organism	14	73.7%
2 organisms	5	26.3%
Wound		
1 organism	4	80.0%
2 organisms	1	20.0%
Trachea		
1 organism	4	66.7%
2 organisms	1	16.7%
3-5 organisms	1	16.7%
Platelets count		
Normal (150-450)	76	56.7%
Thrombocytopenia (<150)	54	40.3%
Thrombocytosis (>450)	4	3.0%
WBCs		
Normal (5000-20000)	80	59.7%
Leukopenia (less than 5000)	41	30.6%
Leukocytosis (more than 20000)	13	9.7%

**Figure 1:**
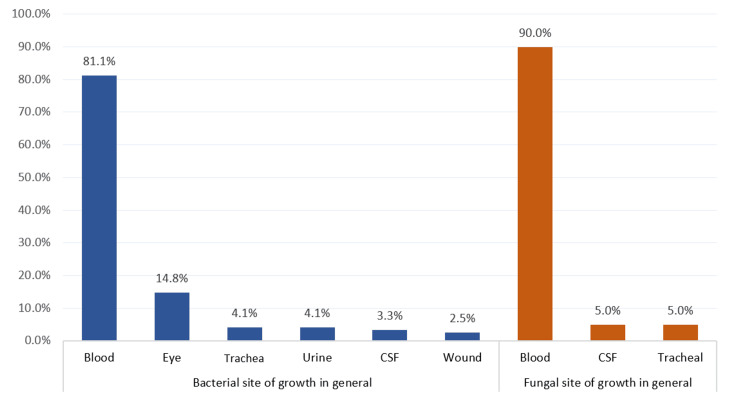
Site of isolated organisms among NICU-admitted neonates with neonatal sepsis, Al-Ahsa, Saudi Arabia (n=134)

Figure 1 shows the site of isolated organisms among NICU-admitted neonates with neonatal sepsis. The most reported isolated bacteria sites were blood (81.1%), eye (14.8%), trachea (4.1%), and urine (4.1%). The most identified isolated fungi sites included blood (90%), CSF (5%), and trachea (5%).

**Figure 2:**
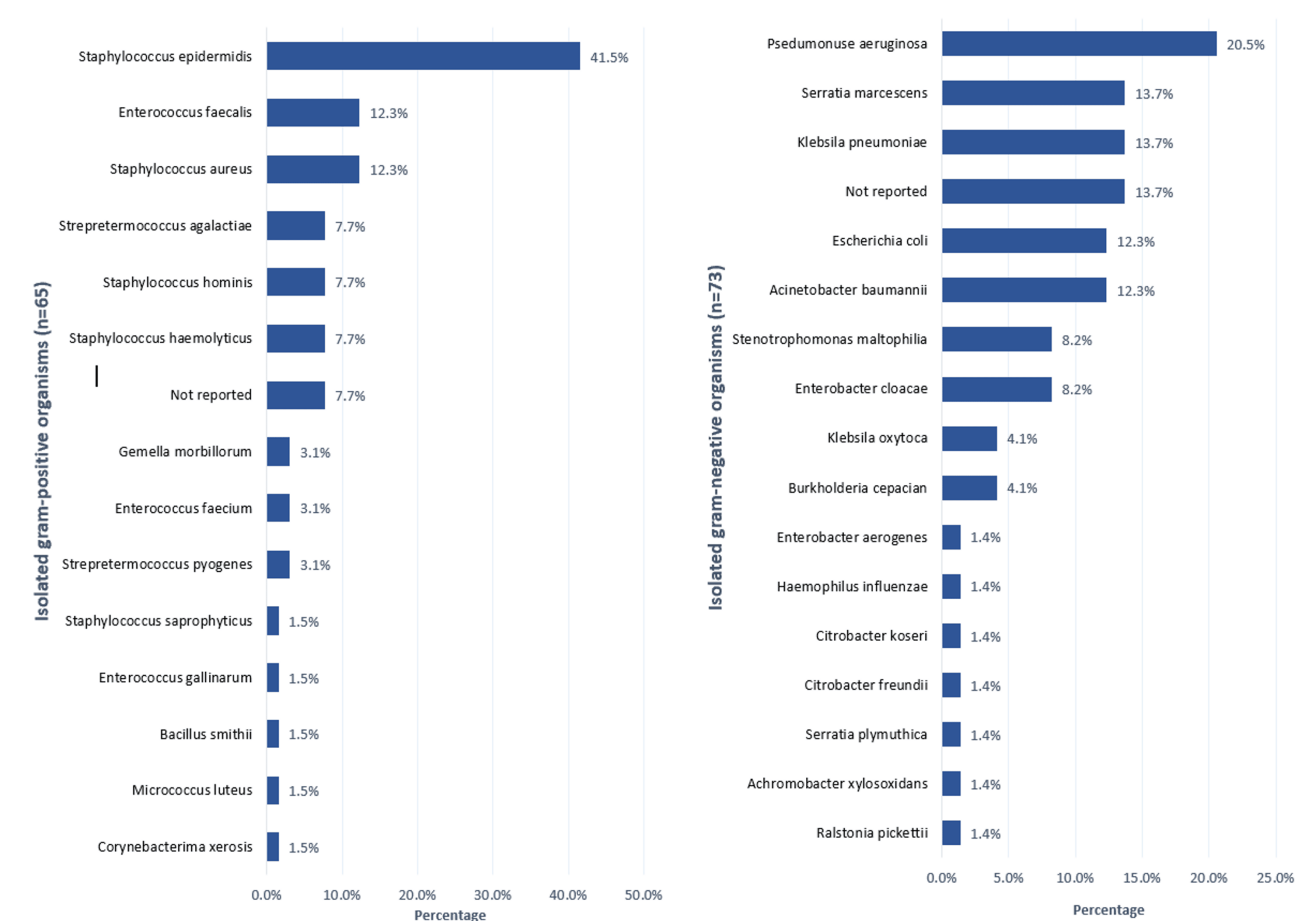
General isolated bacterial organisms among NICU cases with neonatal sepsis

As for the isolated organisms (Figure 2), the most isolated gram-negative bacteria were *Pseudomonas aeruginosa* (20.5%), *Serratia marcescens* (13.7%), *Klebsiella pneumoniae* (13.7%), *E. coli* (12.3%), *Acinetobacter baumannii* (12.3%), and *Stenotrophomonas maltophilia* (8.2%). The least isolated gram-negative bacteria were *Serratia plymuthica* (1.4%), *Achromobacter xylosoxidans* (1.4%), and *Ralstonia pickettii* (1.4%). As for gram-positive bacteria, the most isolated included *Staphylococcus epidermidis* (41.5%), *Enterococcus faecalis* (12.3%), *Staphylococcus aureus* (12.3%), and *Streptococcus agalactiae* (7.7%).

**Figure 3:**
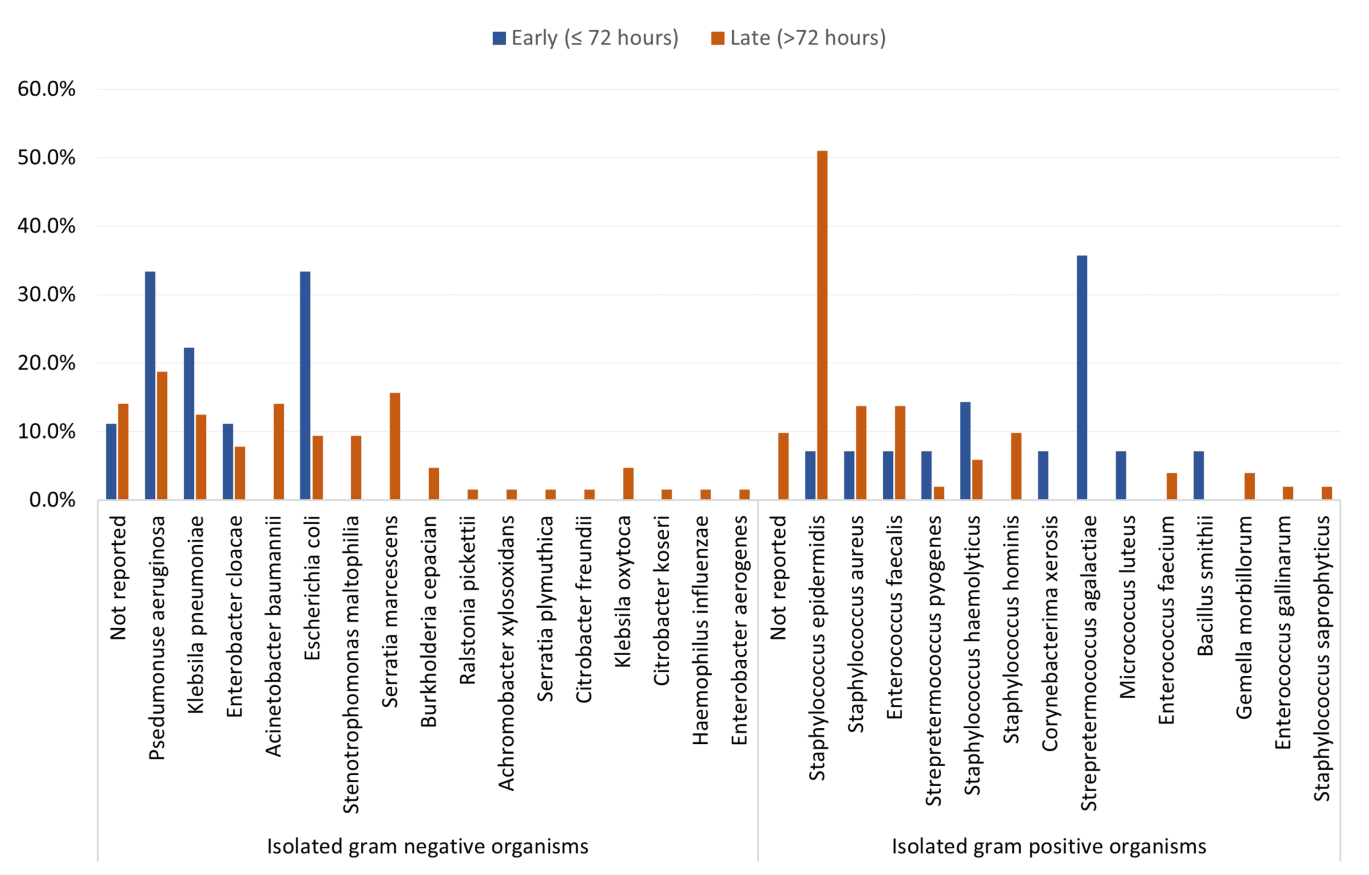
Isolated microorganisms by sepsis onset among study neonates

Figure 3 shows a comparison of isolated gram-negative and gram-positive organisms in neonates with early-onset (≤ 72 hours) and late-onset (> 72 hours) sepsis. As for gram-negative organisms, *Pseudomonas aeruginosa*: This organism is notably more prevalent in early-onset sepsis (33.3%) compared to late-onset (18.8%). *Escherichia coli*: Similar to *Pseudomonas aeruginosa*, *E. coli* is more frequently isolated in early-onset sepsis cases (33.3%) compared to late-onset (9.4%). *Klebsiella pneumoniae*: Shows a higher prevalence in early-onset sepsis (22.2%) compared to late-onset (12.5%). *Serratia marcescens*: Interestingly, this organism is absent in early onset cases but accounts for 15.6% of late-onset sepsis. *Acinetobacter baumannii*: Not isolated in early-onset sepsis but found in 14.1% of late-onset cases. Regarding gram-positive organisms, *Staphylococcus epidermidis* is significantly more common in late-onset sepsis (51.0%) compared to early onset (7.1%); *Staphylococcus aureus* and *Enterococcus faecalis* are both more prevalent in late-onset sepsis (13.7% each) compared to early onset (7.1% each). *Streptococcus agalactiae*: This organism is notably found only in early-onset sepsis cases (35.7%), with no cases reported in late-onset sepsis. *Staphylococcus hominis*: Not present in early-onset sepsis but accounts for 9.8% in late-onset cases. Other observations: overall, gram-negative organisms such as *Acinetobacter baumannii* and *Serratia marcescens* are more common in late-onset sepsis. Gram-positive organisms, especially *Staphylococcus epidermidis*, are significantly more prevalent in late-onset sepsis. *Streptococcus agalactiae* is exclusively found in early-onset sepsis, highlighting its importance in this time frame.

**Figure 4:**
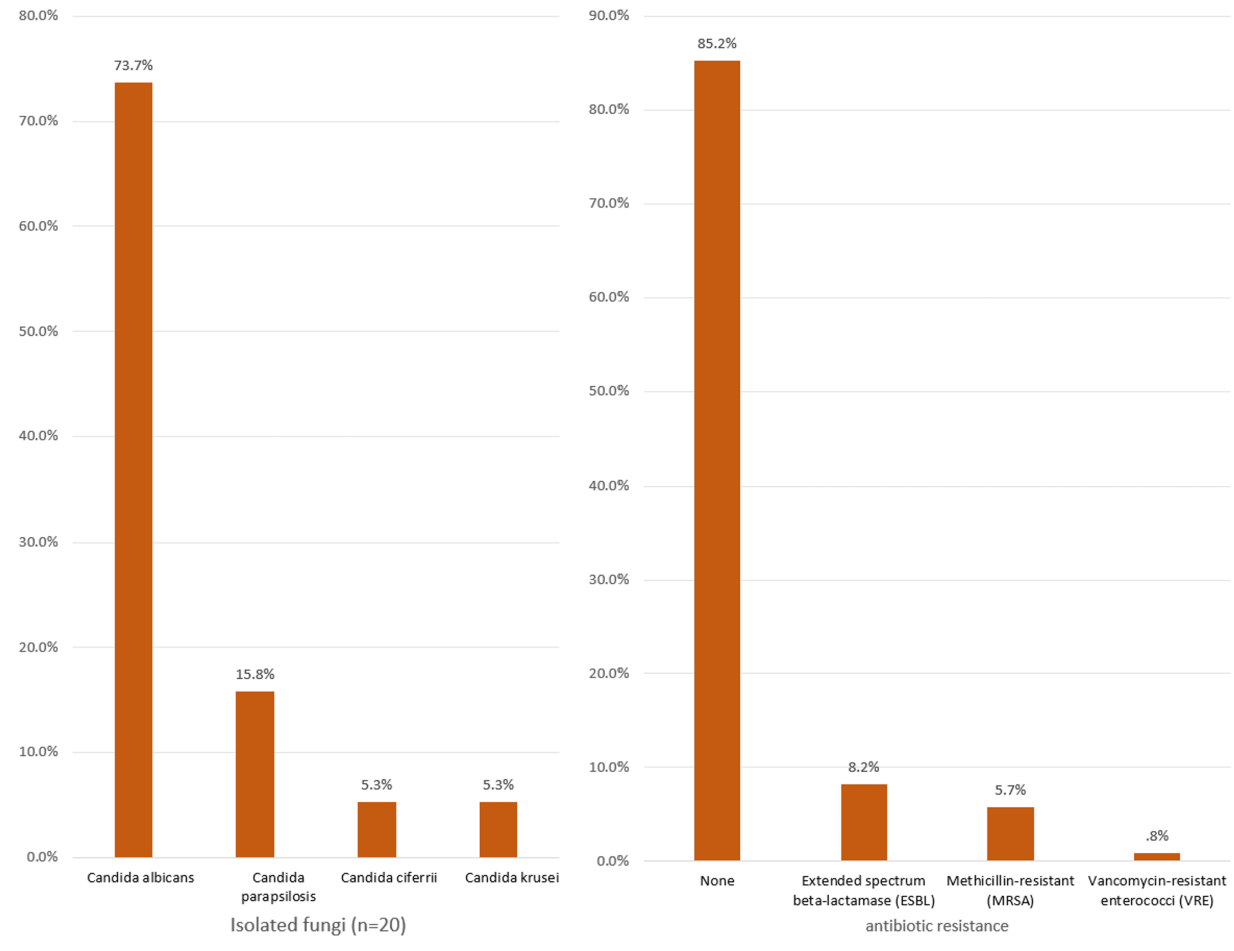
Isolated fungal organisms and antibacterial resistance among NICU cases with neonatal sepsis

Considering fungi, the most isolated were *Candida albicans* (73.7%) (Figure 4). Eighteen (13.4%) cases had resistant bacteria mainly extended-spectrum beta-lactamase (ESBL) (8.2%), methicillin-resistant (MRSA) (5.7%), and vancomycin-resistant enterococci (VRE) (0.8%).

**Figure 5:**
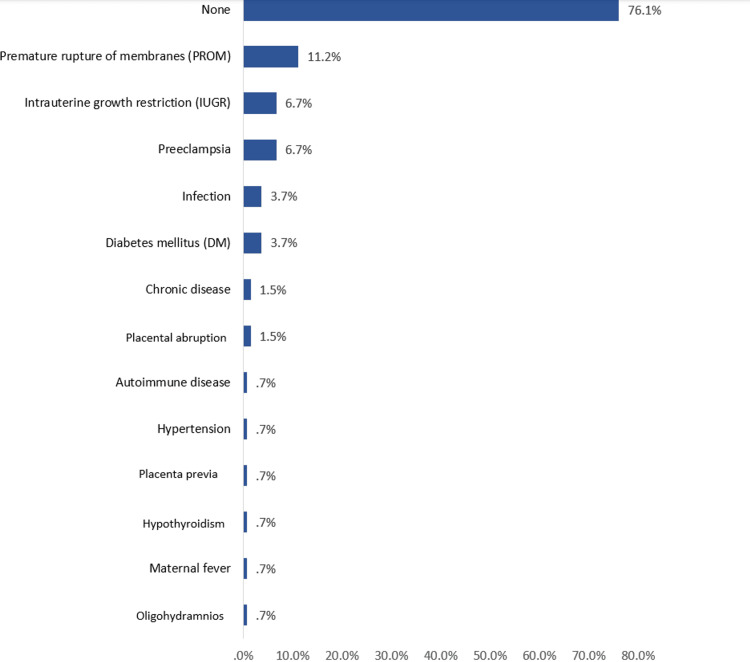
The reported antenatal (maternal) risk factors for neonatal sepsis among NICU cases in Al-Ahsa, Saudi Arabia

As for the reported antenatal (maternal) risk factors for neonatal sepsis, the most identified were PROM (11.2%), IUGR (6.7%), preeclampsia (6.7%), infection (3.7%), and DM (3.7%) (Figure 5).

**Figure 6:**
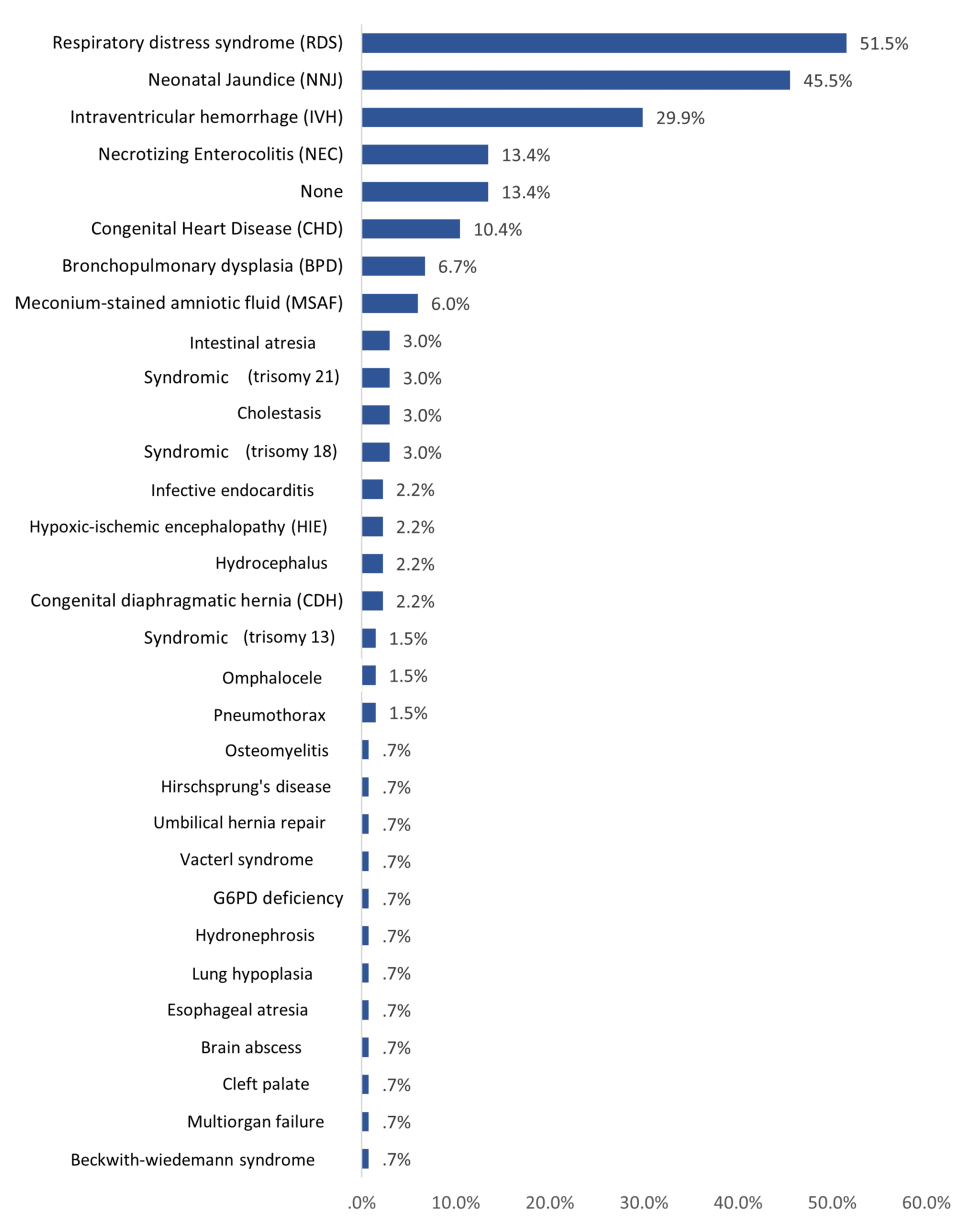
The reported postnatal (neonatal) risk factors for neonatal sepsis among NICU cases in Al-Ahsa, Saudi Arabia

Most of the neonates (76.1%) had no reported maternal risk factors. Regarding postnatal risk factors (neonatal), the most reported were RDS (51.5%), neonatal jaundice (45.5%), intraventricular hemorrhage (29.9%), and necrotizing enterocolitis (13.4%). 13.4% of the neonates had no postnatal risk factors (Figure 6).

**Figure 7:**
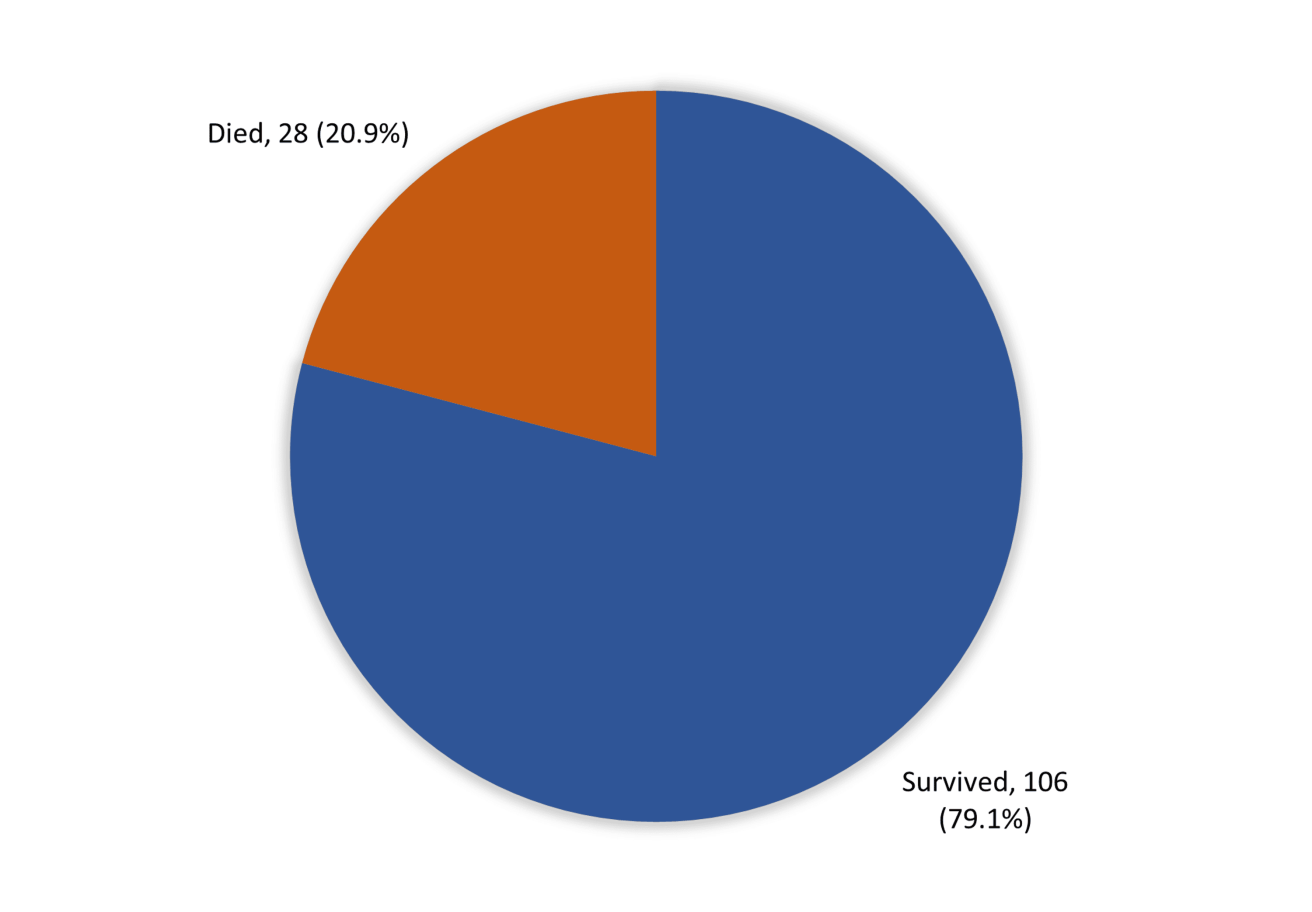
The clinical outcome among NICU admitted neonates with neonatal sepsis, Al-Ahsa, Saudi Arabia (n=134)

The clinical outcome among NICU-admitted neonates with neonatal sepsis. Twenty-eighth (20.9%) of cases died, but most of them (79.1%; 106) survived (Figure 7).

Table 3 presents factors associated with the onset of sepsis among neonates. Gender does not appear to significantly influence the onset of sepsis, with 56.5% of males and 43.5% of females experiencing early onset, compared to 57.7% and 42.3% of males and females in the late-onset group, respectively (p = 0.920). Gestational age shows a significant association with the onset, as more extreme preterm (30.4%) and very preterm (4.3%) neonates experienced early-onset sepsis, while late preterm (16.2%) and full-term neonates (18.9%) had a higher proportion in the late-onset group (p = 0.026). Regarding birth weight, neonates with lower birth weights, such as ELBW (21.7%) and VLBW (13%), had a higher incidence of early-onset sepsis, while neonates with normal birth weight (52.2%) had later-onset sepsis (18.9%) (p = 0.009). Clinical outcomes showed no significant difference between the groups, with 87.0% of early-onset neonates surviving compared to 77.5% in the late-onset group, though 22.5% of late-onset neonates died compared to only 13.0% in the early-onset group (p = 0.309). The number of isolated organisms and infection type (bacterial, fungal, or both) did not show significant differences, with bacterial infections being the most common in both early (95.7%) and late (82.9%) onset cases. Platelet count showed a significant association with thrombocytopenia observed in 21.7% of early-onset and 44.1% of late-onset neonates (p = 0.049). WBC count did not show a significant difference, with similar proportions of leukopenia and leukocytosis in both early and late-onset groups (p = 0.618). 

**Table 3:** Factors associated with onset of sepsis among study neonates P: Pearson X2 test, ^: Exact probability test, * P < 0.05 (significant), ELBW: Extremely low birth weight, VLBW: Very low birth weight, LBW: Low birth weight, NBW: Normal birthweight, WBC: White blood cell

Factor	Onset of sepsis				p-value
	Early (≤ 72 hours)		Late (>72 hours)		
	No	%	No	%	
Gender					.920
Male	13	56.5%	64	57.7%	
Female	10	43.5%	47	42.3%	
Gestation					.289^
Single	23	100.0%	100	90.1%	
Twin	0	0.0%	8	7.2%	
Triple	0	0.0%	3	2.7%	
Gestational age (By weeks)					.026*^
Extreme preterm (<28 W)	7	30.4%	34	30.6%	
Very preterm (28 to <32 W)	1	4.3%	27	24.3%	
Moderate preterm (32 to <34 W)	1	4.3%	11	9.9%	
Late preterm (34 to <37 W)	3	13.0%	18	16.2%	
Full term (37 to 42 W)	11	47.8%	21	18.9%	
Birth weight (By grams)					.009*
ELBW (<1000 g)	5	21.7%	33	29.7%	
VLBW (1000 to 1499 g)	3	13.0%	28	25.2%	
LBW (1500 to 2499 g)	3	13.0%	29	26.1%	
NBW (2500 to 3999 g)	12	52.2%	21	18.9%	
The clinical outcome					.309
Survived	20	87.0%	86	77.5%	
Died	3	13.0%	25	22.5%	
Isolated organism number					.117
Single	20	87.0%	79	71.2%	
Multiple	3	13.0%	32	28.8%	
Type of infection					.262^
Bacterial	22	95.7%	92	82.9%	
Both (Bacterial and Fungal)	0	0.0%	8	7.2%	
Fungal	1	4.3%	11	9.9%	
Type of bacterial organism (Gram stain)					.105
Both (Gram-negative and positive)	1	4.5%	15	15.0%	
Gram-negative	8	36.4%	49	49.0%	
Gram-positive	13	59.1%	36	36.0%	
Platelets count					.049*^
Normal (150-450)	18	78.3%	58	52.3%	
Thrombocytopenia (<150)	5	21.7%	49	44.1%	
Thrombocytosis (>450)	0	0.0%	4	3.6%	
WBCs					.618
Normal (5000-20000)	15	65.2%	65	58.6%	
Leukopenia (less than 5000)	7	30.4%	34	30.6%	
Leukocytosis (more than 20000)	1	4.3%	12	10.8%	

Table 4 outlines the antenatal and postnatal risk factors associated with the onset of sepsis among neonates, categorized into early (≤ 72 hours) and late (> 72 hours) onset. The highest percentage difference in antenatal factors is observed in the "None" category, with 69.6% of early-onset neonates and 77.5% of late-onset neonates having no maternal risk factors, which highlights a trend of more uncomplicated pregnancies in both groups. Among the maternal risk factors, the presence of preeclampsia, intrauterine growth restriction (IUGR), and premature rupture of membranes (PROM) were more common in early-onset sepsis cases, although these differences were not as significant (p = 0.009).

**Table 4:** Antenatal and postnatal risk factors associated with the onset of sepsis among neonates

Risk factors	Onset of sepsis				p-value
	Early (≤ 72 hours)		Late (>72 hours)		
	No	%	No	%	
Antenatal risk factors (Maternal)					.009*
None	16	69.6%	86	77.5%	
Preeclampsia	2	8.7%	7	6.3%	
Intrauterine growth restriction (IUGR)	2	8.7%	7	6.3%	
Premature rupture of membranes (PROM)	4	17.4%	11	9.9%	
Diabetes mellitus (DM)	1	4.3%	4	3.6%	
Abruptio placenta	0	0.0%	2	1.8%	
Oligohydramnios	0	0.0%	1	.9%	
Infection	2	8.7%	3	2.7%	
Maternal fever	1	4.3%	0	0.0%	
Hypothyroidism	0	0.0%	1	.9%	
Placenta previa	0	0.0%	1	.9%	
Chronic disease	2	8.7%	0	0.0%	
Hypertension	1	4.3%	0	0.0%	
Autoimmune disease	1	4.3%	0	0.0%	
Postnatal risk factors (Neonatal)					.001*
None	9	39.1%	9	8.1%	
Intraventricular hemorrhage (IVH)	3	13.0%	37	33.3%	
Respiratory distress syndrome (RDS)	5	21.7%	64	57.7%	
Neonatal jaundice (nnj)	6	26.1%	55	49.5%	
Syndromic (TRISOMY 18)	0	0.0%	4	3.6%	
Congenital heart disease (chd)	0	0.0%	14	12.6%	
Necrotizing enterocolitis (nec)	1	4.3%	17	15.3%	
Bronchopulmonary dysplasia (BPD)	0	0.0%	9	8.1%	
Cholestasis	2	8.7%	2	1.8%	
Congenital diaphragmatic hernia (CDH)	0	0.0%	3	2.7%	
Pneumothorax	0	0.0%	2	1.8%	
BECK-WEITH Diabetes mellitus (DM)	0	0.0%	1	.9%	
Omphalocele	0	0.0%	2	1.8%	
Multiorgan failure	0	0.0%	1	.9%	
Cleft palate	0	0.0%	1	.9%	
Brain abscess	0	0.0%	1	.9%	
Esophageal atresia	0	0.0%	1	.9%	
Meconium-stained amniotic fluid (MSAF)	4	17.4%	4	3.6%	
Lung hypoplasia	0	0.0%	1	.9%	
Hydronephrosis	0	0.0%	1	.9%	
Hydrocephalus	0	0.0%	3	2.7%	
Syndromic (TRISOMY 21)	1	4.3%	3	2.7%	
Syndromic (TRISOMY 13)	0	0.0%	2	1.8%	
G6PD deficiency	1	4.3%	0	0.0%	
VACTERAL syndrome	1	4.3%	0	0.0%	
Hypoxic-ischemic encephalopathy	0	0.0%	3	2.7%	
Infective endocarditis	0	0.0%	3	2.7%	
Intestinal atresia	1	4.3%	3	2.7%	
Umbilical hernia repair	0	0.0%	1	.9%	
Hirschsprung disease	0	0.0%	1	.9%	

In the postnatal risk factors, neonatal conditions like respiratory distress syndrome (RDS) and intraventricular hemorrhage (IVH) showed the most significant differences, with 57.7% of late-onset neonates experiencing RDS, compared to only 21.7% of those with early-onset sepsis (p = 0.001). Similarly, 33.3% of late-onset neonates had IVH, compared to 13.0% in the early-onset group. These findings suggest that neonatal conditions such as RDS and IVH are strongly associated with late-onset sepsis, highlighting the importance of monitoring and managing these factors in neonates. Conversely, conditions like meconium-stained amniotic fluid (MSAF) were more common in early-onset sepsis, with 17.4% in early-onset versus 3.6% in late-onset. These results indicate that both antenatal and postnatal risk factors significantly influence the timing of sepsis onset in neonates, with neonatal complications playing a larger role in late-onset sepsis.

Table 5 shows factors associated with clinical outcomes among NICUs with sepsis. 38.1% of later preterm cases died versus 9.4% of full-term cases (p=.041). Also, 31.6% of cases with ELBW died compared to 9.1% of others with NBW (p=.020). Death was the fate among 44.4% of neonates with critical conditions versus none of the stable cases (p=.001) and among 87.5% of cases with bacterial and fungal infection compared to 14% of others with bacterial infection (p=.001). Likewise, 29.8% of cases with gram-negative bacterial infection died versus 4.1% of others with gram-positive infection (p=.003), and among 40.7% of thrombocytopenia (<150) cases and 46.2% of cases with leukocytosis compared to 11.3% of normal cases (p=.002).

**Table 5:** Factors associated with clinical outcome among study NICUs with sepsis p: Pearson X2 test, ^: Exact probability test,* p < 0.05 (significant), ELBW: Extremely low birth weight, VLBW: Very low birth weight, LBW: Low birth weight, NBW: Normal birthweight

Factors		The clinical outcome				p-value
		Survived		Died		
		No	%	No	%	
Gender	Male	64	83.1%	13	16.9%	.184
	Female	42	73.7%	15	26.3%	
Gestation	Single	96	78.0%	27	22.0%	.544^
	Twin	7	87.5%	1	12.5%	
	Triple	3	100.0%	0	0.0%	
Gestational age (by weeks)	Extreme preterm (<28 W)	29	70.7%	12	29.3%	.041*^
	Very preterm (28 to <32 W)	25	89.3%	3	10.7%	
	Moderate preterm (32 to <34 W)	10	83.3%	2	16.7%	
	Late preterm (34 to <37 W)	13	61.9%	8	38.1%	
	Full term (37 to 42 W)	29	90.6%	3	9.4%	
Birth weight (by grams)	ELBW (<1000 g)	26	68.4%	12	31.6%	.020*
	VLBW (1000 to 1499 g)	28	90.3%	3	9.7%	
	LBW (1500 to 2499 g)	22	68.8%	10	31.3%	
	NBW (2500 to 3999 g)	30	90.9%	3	9.1%	
Mode of delivery	Cesarean section	52	85.2%	9	14.8%	.110
	Vaginal delivery	54	74.0%	19	26.0%	
The clinical condition of the baby	Critical	35	55.6%	28	44.4%	.001*
	Stable	71	100.0%	0	0.0%	
Onset of sepsis	Early (≤ 72 hours)	20	87.0%	3	13.0%	.309^
	Late (>72 hours)	86	77.5%	25	22.5%	
Isolated organism number	Single	82	82.8%	17	17.2%	.071
	Multiple	24	68.6%	11	31.4%	
Type of infection	Bacterial	98	86.0%	16	14.0%	.001*^
	Both (Bacterial and Fungal)	1	12.5%	7	87.5%	
	Fungal	7	58.3%	5	41.7%	
Type of bacterial organism (Gram stain)	Both (Gram-negative and positive)	12	75.0%	4	25.0%	.003*
	Gram-negative	40	70.2%	17	29.8%	
	Gram-positive	47	95.9%	2	4.1%	
Platelets count	Normal (150-450)	71	93.4%	5	6.6%	.001*^
	Thrombocytopenia (<150)	32	59.3%	22	40.7%	
	Thrombocytosis (>450)	3	75.0%	1	25.0%	
WBCs	Normal (5000-20000)	71	88.8%	9	11.3%	.002*
	Leukopenia (less than 5000)	28	68.3%	13	31.7%	
	Leukocytosis (more than 20000)	7	53.8%	6	46.2%	

## Discussion

The current study aimed to assess the pattern and the isolated microorganisms in neonatal sepsis (NS) cases. Also, to identify the most frequent maternal and neonatal risk factors. Neonatal sepsis is a clinical syndrome in a baby who is 28 days of age or younger [24]. Systemic symptoms of infection and the identification of a bacterial pathogen in the bloodstream are its hallmarks. Neonatal sepsis can be further divided into two categories: late-onset neonatal sepsis (LONS) if it develops between days 7 and 28 of life, and early-onset neonatal sepsis (EONS) if the clinical signs of sepsis appear during the first week of life [25].

The current study revealed that most neonates with NS were males with preterm and low birth weight. Also, most cases were delivered normally from a single pregnancy. The vast majority of the neonates had late sepsis (after 72 hours), and about half of the cases were stable.

Regarding isolated organisms, the current study revealed that bacteria were the most isolated organisms (more than three-fourths of the cases) with similar frequency for gram-negative and gram-positive bacteria. Some cases had fungi, and a few cases had both. Blood cultures were the most reported sites for the isolated organisms, but some cases had positive urine cultures, tracheal discharge cultures, and CSF cultures. Also, some isolates from the neonate's eye and wounds were reported. *Staph epidermidis* (G+ve), *Pseudomonas aeruginosa* (G-ve), *Klebsiella pneumoniae* (G-ve), *E. coli* (G-ve), *Enterococcus faecalis* (G+ve), *Staph aureus* (G+ve), and *Acinetobacter baumannii* (G-ve) were the most isolated bacteria. *Candida albicans* was the most isolated fungus among the study neonates. The literature showed similar findings where gram-positive bacteria such as *Streptococcus agalactiae*, *Escherichia coli*, *Staphylococcus aureus*, *Enterococcus*, and *Streptococcus pneumonia* are among those that cause early-onset neonatal sepsis [26]. Gram-negative bacteria, coagulase-negative *Staphylococci* (CONS), *Klebsiella pneumoniae*, and *Acinetobacter baumannii* are among the pathogens that cause late-onset neonatal sepsis [26,27]. Also, the literature showed that fungal causes are uncommon, with the most common fungal cause being *Candida*, which is consistent with the current study findings [28]. Also, CONS, *E. coli*, *S. aureus*, and *Klebsiella* are the most commonly identified bacteria isolates in many other studies in developing countries [29,30]. In Saudi Arabia, Dawodu A et al. [18] found that *Staphylococci* were the major gram-positive isolate occurring in both ‘early’ (≤ 48 h) and ‘late’ (448 h) onset septicemia. Another study by Sobaih BH et al. [19] showed that 10.9% of patients had early-onset sepsis (EOS), and 37.1% had late-onset sepsis (LOS). Both early- and late-onset sepsis were most frequently caused by gram-positive pathogens (74.5% and 87.4%, respectively). In both groups, *Staphylococcus epidermidis* was the most frequently isolated pathogen, which is all very similar to our study findings.

Concerning the associated risk factors of neonatal sepsis, the current study showed neonatal risk factors were more frequent than maternal risk factors. The most reported neonatal risk factors were RDS, neonatal jaundice, intraventricular hemorrhage, and necrotizing enterocolitis. Also, the most reported maternal risk factors were PROM, IUGR, preeclampsia, infection, and DM. Other studies reported similar risk factors and found that maternal risk factors include membrane rupture, chorioamnionitis, and poor prenatal care, while neonatal risk factors include fetal immune system prematurity, congenital dermatologic abnormality, and birth asphyxia [31]. Also, prematurity, PROM, maternal pyrexia, low birth weight, and obstructed labor or birth asphyxia, which is usually characterized by low first and fifth Apgar scores at birth, were reported in the African region [32]. The main risk factors for neonatal sepsis were examined in a 2015 study by A. Doronjski et al. [33] that involved 239 preterm infants in the Neonatal Intensive Care Unit (NICU) and reported that premature rupture of membranes, low gestational age, low birth weight, mechanical ventilation, the implantation of an umbilical venous catheter, and abdominal drainage were the primary risk factors for sepsis. According to MS HASAN et al. [34], prematurity is a risk factor for neonatal sepsis. They found a significant correlation between the onset of sepsis and prematurity. Additionally, it was observed in other studies that supported the idea that sepsis and gestational age are inversely related [18,35]. According to Abdulrahman Al-Matary [20], both neonates with EOS and LOS prematurity were the major neonatal risk factors for sepsis: 16 (48.5%) and 214 (80.8%), respectively.

The mortality rate among the current study cases was about 20%, which is low in comparison to the literature-reported neonatal sepsis-associated mortality rate. Neonatal sepsis, a leading cause of neonatal deaths in developing countries, can cause 52% of case fatalities, contributing to nearly one million deaths and 30-50% of total neonatal deaths, despite its largely preventable nature through prevention, timely recognition, and aggressive supportive care [36].

Limitations

Among the study's limitations were the retrospective design, which could lead to errors or missing data, the relatively small number of blood cultures that tested positive for sepsis, and the high number of patients who had previously received antibiotic treatment. Additionally, the study was single-centered and carried out in one hospital over a one-year period, which limited the external validity of our findings.

## Conclusions

The current study revealed that neonatal sepsis was frequent among the study neonates, mainly late-onset sepsis. Most neonates had LBW and were preterm. Bacterial infection was the most reported, where both gram-positive and gram-negative bacteria were isolated at higher rates in blood samples. Fungal infections were infrequent, and no viruses were isolated. Neonatal risk factors, mainly RDS and neonatal jaundice, were more frequent than maternal risk factors, such as PROM. Nearly one-fifth of the neonates with neonatal sepsis died, which is a low reference to the global trend. Preventive measures with early diagnosis and effective management are crucial to prevent septic shock, multiple organ failure, and death. Early diagnosis allows physicians to identify the cause, enabling more efficient treatment and preventing further complications in neonatal sepsis.
